# Sublingual immunotherapy in children with asthma: A population-based register study

**DOI:** 10.1016/j.jacig.2025.100574

**Published:** 2025-09-30

**Authors:** Jon R. Konradsen, Cecilia Lundholm, Anna M. Hedman, Caroline Stridsman, Hanna Karim, Bronwyn K. Brew, Emma Caffrey Osvald, Samuel Rhedin, Maria Ingemansson, Catarina Almqvist

**Affiliations:** a Department of Women’s and Children’s Health, Karolinska Institutet, Stockholm, Sweden; b Astrid Lindgren’s Children’s Hospital, Karolinska University Hospital, Stockholm, Sweden; c Department of Medical Epidemiology and Biostatistics, Karolinska Institutet, Stockholm, Sweden; d Department of Public Health and Clinical Medicine/The OLIN Unit, Umeå University, Umeå, Sweden; e School of Medicine and Public Health, University of Newcastle, Newcastle, Australia; f Sachs’ Children and Youth Hospital, Stockholm, Sweden

**Keywords:** Allergen-specific immunotherapy, sublingual immunotherapy, asthma, children, epidemiology, spirometry, asthma control, socioeconomic status

## Abstract

**Background:**

Daily sublingual immunotherapy (SLIT) for 3 years reduces symptoms of allergic disease and induces tolerance. Real-life data from children with asthma receiving SLIT are scarce.

**Objective:**

We used population-based data to describe characteristics, SLIT duration, and changes in morbidity in children with asthma prescribed SLIT.

**Methods:**

The study included children (5-17 years) with asthma who were prescribed SLIT, and who were registered in the Swedish National Airway Register (SNAR) before SLIT initiation (N = 1,514), of whom 782 had post-SLIT recordings in the SNAR. Age, sex, Asthma Control Test score (≤19 denotes uncontrolled asthma), spirometry data, number of SLIT tablets dispensed, and socioeconomic background were extracted from the SNAR and other national registers. SLIT duration was classified as <4, ≥4, >12, or >24 months.

**Results:**

SLIT was more common in boys (69%) and adolescents (71%). Most children had parents with higher education (70%), and 25% had uncontrolled asthma. SLIT duration of ≥4, >12, and >24 months was identified in 86%, 50%, and 30%, respectively. Parents with higher education were associated with SLIT duration of ≥4 months (odds ratio = 3.69; 95% confidence interval 2.75-4.96). SLIT duration of >12 months was associated with lower risk of post-SLIT uncontrolled asthma (adjusted odds ratio = 0.49; 95% confidence interval, 0.25-0.99). No associations were found between SLIT duration of ≥4, >12, or >24 months and changes in spirometry.

**Conclusion:**

Longer SLIT duration was associated with higher education in parents and lower risk of uncontrolled asthma. Measures to improve persistence in SLIT in children with asthma may have important clinical implications.

Asthma is characterized by airway inflammation, variable airflow obstruction, and respiratory symptoms, and is one of the most common chronic conditions in children.^1^ Up to 60% of children with asthma are allergic, and in these patients, symptoms are often triggered by environmental allergens such as pollen and house dust mites.^2^ Clinical guidelines recommend a stepwise approach to treatment with bronchodilators, anti-inflammatory and biological drugs, and allergen immunotherapy (AIT) for adult patients.^3^

AIT targets the immunologic cause of asthma and rhinitis—airborne allergens—and has the potential to alter the natural course of allergic disease.^4^ Subcutaneous immunotherapy to grass and birch pollen has long been available, whereas sublingual immunotherapy (SLIT) tablets, a more convenient way to take the medication, were introduced more recently.^5^ SLIT induces tolerance and reduces symptoms of allergic disease through daily sublingual exposure (tablet or liquid) to allergens. At the time of the study, SLIT tablets were approved in Sweden for the treatment of allergic rhinitis in patients from age 5 years (grass; Grazax; phleum pratense), 12 years (house dust mites; Acarizax; dermatophagoides pteronyssinus and dermatophagoides farinae), and 18 years (birch; Itulazax, betula verrucosa), manufactured by ALK-Nordic A/S.^4^ However, real-life data on sociodemographic and clinical characteristics of children with asthma who initiate SLIT are scarce.

Clinical effects of SLIT are observed after 4 months of treatment,^5^ but continued SLIT up to 3 years has been proposed for persistent effects.^6^, ^7^, ^8^ Persistence in SLIT is a major challenge, as the tablets must be taken daily. A previous real-life study including adult patients showed that only 7% of SLIT patients completed 3 years of treatment, with the median SLIT duration being 6 months.^9^ Associations with discontinued treatment include lower socioeconomic status (SES) and younger age,^9^^,^^10^ but few studies have investigated actual SLIT duration and factors associated with discontinued treatment in child populations.

SLIT is currently not included in clinical guidelines for the treatment of asthma in children, but it is an established treatment option for patients with rhinitis and allergy to pollen or house dust mites.^11^ Up to 80% of children prescribed SLIT for rhinitis also have asthma,^12^ but uncontrolled asthma is a contraindication for SLIT because of the risk of adverse effects.^3^ There is a lack of evidence regarding the effect of SLIT on standardized asthma outcomes such as symptom scores and pulmonary function measurements. Two independent systematic reviews concluded that the addition of SLIT to standard asthma treatment could reduce the risk of asthma exacerbations and symptoms, but the results were uncertain and hampered by the use of nonstandardized asthma outcomes.^12^^,^^13^ A recent register-based study demonstrated that SLIT liquid prevented intensification of asthma treatment and asthma onset.^14^

The purpose of this study was to use real-life, population-based data from children with asthma to expand the knowledge regarding characteristics of those prescribed SLIT, duration of SLIT, and effects of SLIT on standardized asthma outcomes (Asthma Control Test [ACT] score and pulmonary function measurements). Specifically, we aimed to (1) describe sociodemographic and clinical characteristics of children who initiated SLIT (Grazax, Itulazax, or Acarizax), (2) assess SLIT duration and factors associated with SLIT duration of >4 months, and (3) explore associations between SLIT duration (>4, >12, and >24 months) and posttreatment changes in asthma morbidity.

## Methods

### Study design, data sources, linkage, and ethics

This was a population-based cohort study of children with asthma who were prescribed SLIT. The subjects were identified from the Swedish National Airway Register (SNAR), which was initiated in 2013 and comprises data on patients with a physician diagnosis of asthma from primary and secondary care (International Classification of Diseases version 10 code J45).^15^ The diagnosis of asthma in SNAR is made by the treating physician. Registrations in the SNAR can be made by clinic staff at each health care visit for asthma and include data on age, sex, ACT score, and pulmonary function measurements. Unique personal identification numbers^16^ were used to link data from the SNAR to the Swedish Prescribed Drug Register (SPDR)^17^ and the Longitudinal Integrated Database for Health Insurance and Labour Market Studies (LISA).^18^ Ethical approval was obtained from the Swedish Ethical Review Authority (approvals 2018/1697-31/1 and 2023-03916-02), and informed consent was waived because of the register-based nature of the study.

### Study population

**Fig 1:**
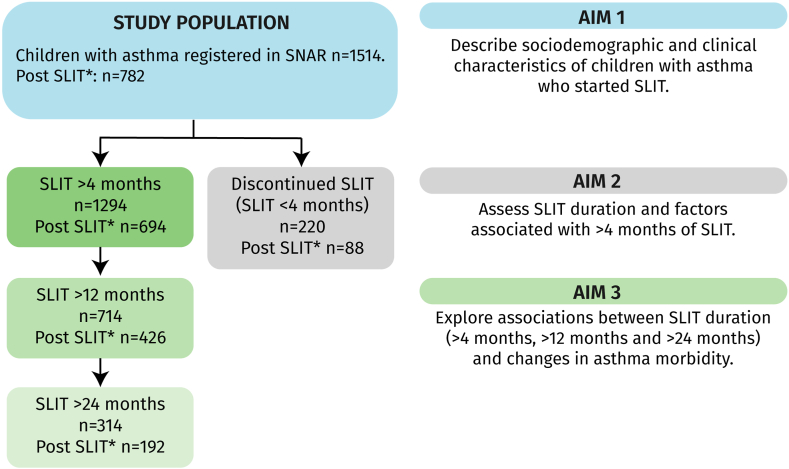
Flowchart demonstrating patient inclusion, SLIT duration, and study aims. ∗Post-SLIT assessment; clinical data from SNAR.

The study included all children (5-17 years) with asthma treated with SLIT and registered in the SNAR before SLIT initiation (Fig 1). Dispensed SLIT in the SPDR was defined as occurrence of the Anatomical Therapeutic Chemical code for Grazax (V01AA02), Itulazax (V01AA05), or Acarizax (V01AA03) in the period from January 1, 2013, to December 31, 2023 (N = 1,514). Information was retrieved from the health care visit registered in the SNAR before SLIT initiation. For patients with multiple health care visits, we used clinical data from the most recent visit before SLIT initiation. Post-SLIT health care visits recorded in the SNAR were identified in 782 of the 1,514 children included in the study.

### Variables and definitions

For SLIT initiation, we used the date when a child was prescribed and dispensed Grazax, Itulazax, and/or Acarizax in the study period from July 1, 2006, to December 31, 2023. Tablets are prescribed as packages of either 30 tablets (1 month’s treatment), 90 tablets (3 months’ treatment), or 30 + 90 tablets (4 months’ treatment).

Treatment duration was classified as <4 or ≥4 months in children with ≥1 dispensed prescription of Grazax, Itulazax, or Acarizax. More than one dispensation corresponds to SLIT duration of ≥4 months. SLIT durations of >12 and >24 months were classified according to the number of tablets in the dispensed prescriptions in the SPDR. Children who initiated SLIT after January 1, 2023 (n = 86), and January 1, 2022 (n = 471), were excluded from analyses of durations of >12 and >24 months, respectively.

### Sociodemographic variables

Sex was classified as male or female. Age at SLIT initiation was classified in age groups: younger child (5-11 years) or adolescent (12-17 years). Socioeconomic background was classified as high or low using data on parental education at treatment start from the LISA. High socioeconomic background was used if at least one parent had a university education and labeled as parent having higher education.

### Clinical variables

Asthma control was assessed using validated questionnaires: the ACT for children aged ≥12 years, and the childhood ACT for children aged 5-11 years. A score of ≤19 in either questionnaire denotes uncontrolled asthma, with better control indicated by higher values.^19^

Prebronchodilator forced expiratory volume in 1 second (FEV_1_) was reported using percentages of predicted values on the basis of reference values from Solymar.^20^ The ratio of FEV_1_ to forced vital capacity (FVC) was calculated.

Bronchodilator response (BDR) was reported in % and calculated in accordance with Global Initiative for Asthma guidelines as follows:^3^ {[postbronchodilator (L) − prebronchodilator (L)]/[prebronchodilator (L)]} × 100 mL.

Fractional exhaled nitric oxide (Feno) was reported in parts per billion.

### Statistical analyses

Descriptive data for all aims were presented as numbers (%) for sex, age group, parent with higher education, uncontrolled asthma, SLIT durations of <4, >4, >12, and >24 months, and means with standard deviations (SDs) for FEV_1_%, FEV_1_/FVC, BDR, and Feno. Data were further stratified by type of SLIT treatment: Grazax, Itulazax, or Acarizax.

To assess associations between background factors and SLIT duration of >4 months (aim 2), we performed logistic regression with SLIT duration of >4 months as outcome and sex, age group, parent with higher education, and pretreatment percentage predicted FEV_1_%, FEV_1_/FVC, BDR, and Feno as predictors.

To explore associations and changes in asthma morbidity related to SLIT duration (aim 3), the predictors were SLIT duration of <4 months versus treatment duration of >4, >12, and >24 months. Logistic regression analyses were performed with uncontrolled asthma as the outcome, and linear regression was performed to assess associations with the outcome variables of posttreatment ACT score, FEV_1_%, FEV_1_/FVC, BDR, and Feno. The regression analyses were performed both for any SLIT and stratified by type of treatment. Analyses were performed with and without adjustment for potential confounders identified *a priori,* as indicated in the tables. Exposure age group and sex were not considered to be influenced by confounders, so only crude results are presented for these associations. The results of the regression analyses are presented as odds ratios (ORs) or regression coefficients (beta) with 95% confidence intervals. All statistical analyses were conducted by STATA BE 18.0 (StataCorp), and *P* < .05 was considered statistically significant.

## Results

The cohort consisted of 1,514 children with asthma who were prescribed SLIT (Grazax, Itulazax, and/or Acarizax) and who were registered in the SNAR before the first SLIT tablets were dispensed (Fig 1 and Table I). SLIT was more common in boys (69%) than girls (31%). Grazax was the most prevalent prescribed treatment (1,026, 67.8%), followed by Itulazax (489, 32.3%), and Acarizax (291, 19.2%). All 3 treatments were more common in adolescents (71.1%) than younger children (28.9%), and the largest difference between age groups was seen for Itulazax (16.3% younger children and 83.8% adolescents). Most children who initiated SLIT had parents with higher education, with slightly fewer in those receiving Acarizax. Uncontrolled asthma was observed in 24.7% of all children who initiated SLIT. Pre-SLIT asthma control, lung function, and Feno are shown in Table I and were similar between treatment groups.

**Table I:** Sociodemographic and clinical characteristics

Characteristic	No.	SLIT initiaton (N = 1,514)	Grazax initiation (n = 1,026)	Itulazax initiation (n = 489)	Acarizax initiation (n = 291)
Female, no. (%)	1,514	470 (31.0)	311 (30.3)	170 (34.8)	90 (30.9)
Male, no. (%)	1,514	1,044 (69.0)	715 (69.7)	319 (65.2)	201 (69.1)
Younger child, no. (%)	1,514	438 (28.9)	350 (34.3)	78 (16.3)	75 (25.8)
Adolescent, no. (%)	1,514	1,076 (71.1.0)	672 (65.8)	402 (83.8)	216 (74.2)
Parent with higher education, no. (%)	1,514	1,060 (70.0)	727 (70.9)	360 (73.6)	184 (63.2)
No parent with higher education, no. (%)	1,514	454 (30.0)	299 (29.1)	129 (26.4)	107 (36.8)
ACT score, mean (SD)	954	21.3 (3.6)	21.4 (3.7)	21.3 (3.7)	21.2 (3.6)
ACT score ≤ 19, no. (%)	954	236 (24.7)	162 (25.2)	85 (26.4)	41 (22.3)
ACT score > 19, no. (%)	954	718 (75.3)	480 (74.8)	238 (74.6)	143 (77.7)
FEV_1_ (%), mean (SD)	783	91.1 (12.6)	91.3 (12.2)	92.8 (12.7)	90.3 (13.0)
FEV_1_/FVC, mean (SD)	714	0.85 (0.07)	0.86 (0.07)	0.85 (0.07)	0.84 (0.07)
BDR FEV_1_ (%), mean (SD)	556	4.3 (8.1)	4.2 (7.9)	2.8 (8.2)	5.2 (7.8)
Feno, ppb, mean (SD)	479	22.0 (23.5)	20.6 (22.3)	20.0 (17.8)	32.1 (31.5)

The mean (SD) duration between pre- and post-SLIT recordings in SNAR was 21.2 (16.8) months. Characteristics of children with post-SLIT clinical recordings in SNAR (n = 782) are presented in Table E1 in this article’s Online Repository available at www.jaci-global.org. Of these, 624 had clinical data from pre-SLIT recordings in SNAR.

### SLIT duration

SLIT duration of <4 months was identified in 14.5% of children, and 85.5% had a SLIT duration of >4 months, 50.0% of >12 months, and 30.1% of >24 months (Fig 1 and Table II). The prevalences of SLIT duration of <4 months were similar between Grazax, Itulazax, and Acarizax. The mean (SD) treatment duration was 1.2 (0.9) years for both Grazax and Acarizax and 0.9 (0.7) years for Itulazax.

**Table II:** SLIT duration, stratified by sex and age group

Characteristic	All	Boy	Girl	Age 5-11 years	Age 12-17 years∗
SLIT initiation	1,514	1,044	470	438	1,076
SLIT duration					
<4 months	220 (14.5)	152 (14.6)	68 (14.5)	54 (12.3)	166 (15.4)
≥4 months	1,294 (85.5)	892 (85.4)	402 (85.5)	384 (87.7)	910 (84.6)
>12 months	1428	984	444	412	1016
>12 months	714 (50.0)	504 (51.2)	210 (47.3)	241 (58.5)	473 (46.6)
>24 months	1043	727	316	308	735
>24 months	314 (30.1)	220 (30.3)	94 (29.8)	119 (38.6)	195 (26.5)
Grazax initiation	1,026	715	311	350	672
Grazax duration					
<4 months	153 (14.9)	95 (13.2)	58 (18.7)	44 (12.6)	106 (15.8)
≥4 months	873 (85.1)	620 (86.7)	253 (81.4)	306 (87.4)	566 (84.2)
>12 months	956	670	286	326	629
>12 months	506 (52.9)	367 (54.8)	139 (48.6)	193 (59.2)	313 (49.8)
>24 months	711	500	211	246	465
>24 months	234 (32.9)	169 (33.8)	65 (30.8)	102 (41.5)	132 (28.4)
Itulazax initiation	489	319	170	78	402
Itulazax duration					
<4 months	74 (15.1)	45 (14.1)	29 (17.1)	7 (9.0)	65 (16.2)
≥4 months	415 (84.9)	274 (85.9)	141 (82.9)	71 (91.0)	337 (83.8)
>12 months	475	310	165	77	390
>12 months	185 (38.9)	125 (40.3)	60 (36.4)	39 (50.6)	142 (36.4)
>24 months	293	199	94	49	239
>24 months	46 (15.7)	27 (13.6)	19 (20.2)	10 (20.4)	35 (14.6)
Acarizax initiation	291	201	90	75	216
Acarizax duration					
<4 months	51 (17.5)	36 (17.9)	15 (16.7)	8 (10.7)	43 (19.9)
≥4 months	240 (82.5)	165 (82.1)	75 (83.3)	67 (89.3)	173 (80.1)
>12 months	264	182	82	67	197
>12 months	138 (52.3)	94 (51.7)	44 (53.7)	42 (62.7)	96 (48.7)
>24 months	209	141	68	55	154
>24 months	68 (32.5)	48 (34.0)	20 (29.4)	24 (43.6)	44 (28.6)

Data are presented as nos. or as nos. (%). All column percentages are within each group. Children who initiated SLIT after January 1, 2023 (n = 86), and January 1, 2022 (n = 471), were excluded from analyses because of durations of >12 and >24 months, respectively.

∗ Four individuals initiated Grazax treatment at age 18 and were included because they received their first SLIT treatment (Itulazax) at age 17. Similarly, 9 individuals received Grazax before age 18 but initiated Itulazax after turning 18.

### Sociodemographic and clinical factors associated with SLIT duration of >4 months

SLIT duration of >4 months was more common in younger children than adolescents, but the difference was not statistically significant (Table II). More patients with SLIT duration of >4 months had parents with higher education compared with patients with duration of <4 months (Table III). There was thus an association between parents with higher education and SLIT duration of >4 months (OR = 3.69; 95% confidence interval, 2.75-4.95), whereas no significant associations were found between sex, age group, pretreatment asthma control, FEV_1_%, FEV_1_/FVC, BDR, or Feno and SLIT duration of >4 months.

**Table III:** Univariate comparisons and logistic regression analysis

Characteristic	Univariate comparisons		Logistical regression			
			Crude		Adjusted∗	
	SLIT duration of <4 months (n = 220)	SLIT duration of >4 months (n = 1,294)	No.	OR_crude_	No.	OR_adj_
Female sex, no. (%)	68 (30.9)	402 (31.1)	1,514	1.01 (0.73-1.37)	—	—
Adolescent, no. (%)	166 (75.5)	910 (70.3)	1,514	0.77 (0.55-1.07)	—	—
Parent with higher education, no. (%)	97 (44.1)	963 (74.4)	1,514	3.69 (2.75-4.96)	—	—
ACT score, mean (SD)	21.1 (3.6)	21.4 (3.6)	954	1.02 (0.98-1.07)	954	1.02 (0.96-1.07)
ACT score ≤ 19, no. (%)	37 (25.9)	199 (24.5)	954	0.93 (0.62-1.40)	954	1.00 (0.66-1.53)
FEV_1_ (%), mean (SD)	92.3 (12.5)	90.9 (12.6)	783	0.99 (0.98-1.01)	665	0.99 (0.98-1.01)
FEV_1_/FVC, mean (SD)	0.85 (0.07)	0.85 (0.07)	714	1.82 (0.10-32.6)	603	1.32 (0.06-27.6)
BDR FEV_1_ (%), mean (SD)	4.3 (5.8)	4.2 (8.5)	556	1.00 (0.97-1.03)	464	0.99 (0.97-1.02)
Feno (ppb), mean (SD)	21.5 (18.2)	22.0 (24.3)	479	1.00 (0.99-1.01)	394	1.00 (0.99-1.02)

Shown is association between sociodemographic and pretreatment clinical characteristics with SLIT duration of >4 months vs SLIT duration of <4 months, adjusted for potential confounders.

∗ No confounders were identified for sex, age, or parental education. ACT was adjusted for sex and parental education. FEV_1_%, FEV_1_/FVC, and BDR were adjusted for parental education. Feno was adjusted for sex, age group, and parental education.

### Associations between SLIT duration and posttreatment clinical outcomes

SLIT duration of >12 months was less likely to be associated with posttreatment uncontrolled asthma (OR_adj_ = 0.49; 95% confidence interval, 0.25-0.99), whereas no significant associations were observed for SLIT duration of >4 or >24 months (Table IV). No associations were found between SLIT duration of >4, >12, or >24 months and posttreatment ACT score, FEV_1_%, FEV_1_/FVC, BDR, or Feno (Table IV).

**Table IV:** Associations between exposure treatment duration and outcome posttreatment clinical characteristics

Characteristic	Treatment duration								
	>4 months vs <4 months			>12 months vs <4 months			>24 months vs <4 months		
	No.	OR_crude_	OR_adj_	No.	OR_crude_	OR_adj_	No.	OR_crude_	OR_adj_
Uncontrolled asthma	542	0.64 (0.34 to 1.21)	0.65 (0.34 to 1.24)	357	0.49 (0.25 to 0.97)	0.49 (0.25 to 0.99)	203	0.56 (0.26 to 1.16)	0.52 (0.24 to 1.14)

	**No.**	**Beta_crude_**	**Beta_adj_**	**No.**	**Beta_crude_**	**Beta_adj_**	**No.**	**Beta_crude_**	**Beta_adj_**

ACT score, mean (SD)	542	−0.15 (−1.1 to 0.79)	−0.20 (−1.2 to 0.76)	357	0.10 (−0.9 to 1.1)	0.13 (−0.9 to 1.1)	203	0.05 (−1.0 to 1.1)	0.09 (−1.0 to 1.2)
FEV_1_ (%), mean (SD)	367	1.86 (−2.3 to 6.1)	2.26 (−2.4 to 6.9)	266	1.08 (−3.4 to 5.6)	1.58 (−4.0 to 7.2)	157	0.61 (−3.8 to 5.0)	−0.54 (−4.8 to 3.7)
FEV_1_/FVC, mean (SD)	335	0.02 (0.0 to 0.1)	0.02 (0.0 to 0.1)	236	0.02 (0.0 to 0.1)	0.02 (0.0 to 0.1)	137	0.02 (0.0 to 0.1)	0.01 (0.0 to 0.1)
BDR FEV_1_ (%), mean (SD)	258	−6.23 (−17.1 to 4.6)	−5.62 (−15.3 to 4.0)	183	−6.31 (−17.2 to 4.6)	−5.34 (−14.2 to 3.2)	110	−5.98 (−17.1 to 5.1)	−4.3 (−12.4 to 3.8)
Feno (ppb), mean (SD)	245	−7.3 (−18.4 to 3.8)	−6.99 (−17.3 to 3.3)	169	−8.18 (−19.5 to 3.1)	−7.3 (−17.6 to 3.0)	90	−8.69 (−20.4 to 3.0)	−7.9 (−17.4 to 1.6)

Uncontrolled asthma and ACT were adjusted for sex and parental education. FEV_1_, FEV_1_/FVC, and BDR were adjusted for parental education; Feno was adjusted for sex, age group, and parental education.

In analyses stratified by treatment type, Grazax duration of >12 months (OR_adj_ = 0.46, 0.22-0.96) and Acarizax duration of >4 months (OR_adj_ = 0.35, 0.14-0.87) and >12 months (OR_adj_ = 0.23, 0.08-0.80) were all less likely to be associated with posttreatment uncontrolled asthma (Table E1).

### Pre- and posttreatment changes in asthma morbidity related to SLIT duration

The proportion of children with uncontrolled asthma was reduced by 0, 6.5%, 11.7%, and 10.1% among children with treatment durations of <4, >4, >12, and >24 months, respectively (Table V). The mean increases in Feno were 11.6, 2.3, 2.3, and 3.9 ppb in children with SLIT duration of <4, >4, >12, and >24 months, respectively. Pre- to posttreatment changes in ACT score, FEV_1_%, FEV_1_/FVC, and BDR were similar in children with SLIT duration of <4, >4, >12, and >24 months.

**Table V:** Comparison of differences before and after treatment for children by treatment duration

Characteristic	Treatment duration						
	<4 months	>4 months		>12 months		>24 months	
	Change	Mean change	*P*	Change	*P*	Change	*P*
Uncontrolled asthma (%)	0.0	−6.5	<.001	−11.7	<.001	−10.1	.20
ACT score	0.3	0.3	.88	0.5	.79	0.3	.97
FEV_1_ (%)	3.2	2.8	.88	3.1	.99	2.4	.63
FEV_1_/FVC	0.00	0.00	.97	−0.01	.83	−0.01	.55
BDR FEV_1_ (%)	0.0	−0.6	.78	−1.6	.53	−0.4	.87
Feno (ppb)	11.6	2.3	.053	2.3	.08	3.9	.18

*Change* refers to posttreatment observations compared to pretreatment observations in children with treatment durations of <4, >4, >12, and >24 months. Changes in uncontrolled asthma were calculated as: [pretreatment uncontrolled asthma (%) − posttreatment uncontrolled asthma (%)]. Changes in ACT, FEV_1_, FEV_1_/FVC, BDR FEV_1_, and Feno were calculated as: [mean of posttreatment observations − mean of pretreatment observations in children with treatment duration <4, >4, >12, and >24 months]. *P* values refer to comparison with treatment duration of <4 months.

## Discussion

In this study, we used population-based data on children (5-17 years) with asthma to expand the real-life knowledge regarding children prescribed SLIT. We found that SLIT was most common in boys and adolescents, Grazax was the most common treatment prescribed, and most children receiving SLIT had parents with higher education. Furthermore, we found that only a minority of children had SLIT duration of >2 years and that SLIT duration of >4 months was associated with having parents with higher education. Last, we found that SLIT duration of >12 months was inversely associated with uncontrolled asthma and that the proportion with uncontrolled asthma was reduced in children with SLIT duration of >4 months. There were no associations between SLIT duration, mean ACT scores, or spirometry findings.

In our study, 69% of the patients were boys and 31% were girls. This finding is similar to what has been reported in a previous real-life study on SLIT patients from Sweden and Denmark.^21^ Male sex is strongly associated with IgE sensitization to airborne allergens from early childhood up to young adulthood.^22^ Thus, boys are most likely to experience from allergic disease and be offered SLIT, thus explaining our findings. Most study participants were adolescents (71%), which is to be expected because only Grazax is approved for children aged <12 years. Acarizax is approved from age 12 years and Itulazax from age 18 years.^4^ It is noteworthy that a large proportion of the study population was prescribed SLIT while the patients were under the approved age limits. We speculate that this finding reflects that clinicians consider SLIT to be safe and effective also in younger children with severe allergies. Grazax was the most common treatment in this cohort, which could be related to both the age limits and the fact that Grazax has been available on the market since 2006, whereas Acarizax was approved in 2015 and Itulazax in 2019 in Sweden.

Most children prescribed SLIT had parents with higher education. SLIT duration of >4 months was associated with having parents with higher education. These findings are in line with results from a Danish register-based study on both children and adults in which AIT was less common in patients with lower education.^10^ The relationship between asthma, allergy, and SES is complex, as low SES has been associated with asthma,^23^ but allergies are positively associated with SES.^24^ Thus, the large proportion of parents with higher education in this cohort may be explained by the fact that allergies are more common in families with high SES. Another, more speculative, explanation is that well-educated parents could be more likely to know about AIT and ask for SLIT from physicians and may be more motivated to ensure continual treatment in their children. In Sweden, families do not incur any costs for SLIT to children, so financial considerations are not likely to explain our findings.

The prevalence of uncontrolled asthma in our study is lower than what has been previously observed in pediatric asthma cohorts using other definitions of uncontrolled asthma.^25^ We speculate that this difference may be due to the inclusion criteria of our study, which only involves children prescribed SLIT. Because uncontrolled asthma is a contraindication for SLIT, this could explain the lower prevalence observed.^26^ Nevertheless, we found that almost a quarter of patients had uncontrolled asthma before SLIT. This is a novel finding, but it should be emphasized that asthma treatment may have been intensified after the health care visit and before initiating SLIT, so the asthma was not necessarily uncontrolled at the time of SLIT initiation.

We found that 85% continued SLIT for >4 months, and only 30% had a SLIT duration of >24 months. Several studies have shown poor persistence in SLIT.^27^, ^28^, ^29^, ^30^ Our findings show shorter SLIT duration than in an open-label noninterventional Nordic study of 392 SLIT patients, in which 55% completed a 3-year treatment period and 37% stopped before the end of the study.^21^ However, in a retrospective analysis of a community pharmacy database from the Netherlands (N = 6,486), only 7% of SLIT patients (adults and children) were treated for 3 years.^9^ Another retrospective study of 142 SLIT patients showed that 46% completed their treatment.^31^ Thus, our results add to evidence that poor persistence to the treatment plan is a major problem in children with asthma prescribed SLIT. Clinical effects of SLIT are often experienced after 4 months of treatment,^5^ and patients might be less motivated to continue treatment because they already experienced an effect, possibly explaining these findings. However, these short treatment periods may not result in sustained effects. Sustained immunologic and clinical effects after SLIT termination requires 3 years of treatment; 2 years of SLIT have been shown to be insufficient for persistent clinical effects.^8^^,^^32^ Thus, it is crucial to facilitate SLIT persistence. Factors that might improve persistence include increasing patient knowledge about the disease and SLIT, and strengthening the patient– or parent–doctor partnership.^33^

Previous studies of the clinical effects of SLIT in relation to asthma have shown less frequent asthma onset, slower asthma progression, and reduced risk of asthma exacerbations and symptoms.^12^^,^^14^^,^^34^^,^^35^ Our study adds to these results by using standardized questionnaires to show that SLIT duration of >12 months was inversely associated with uncontrolled asthma and that the proportion of children with uncontrolled asthma was reduced after treatment among children with SLIT duration of >4 months. Similar estimates were observed for patients with >2 years of SLIT. However, these estimates did not reach statistical significance, likely because of the small number of patients with a SLIT duration of >24 months. We observed different effects for Itulazax compared to Grazax and Acarizax, which could be explained by exposure to other tree-pollen allergens, or it could simply be due to the smaller study population. Notably, many factors can influence the ACT score, such as receipt of controller medication, adherence, and environmental exposure, including pollen season, and we have not adjusted for these factors in our analyses.

We could not demonstrate any associations between lung function measurements and SLIT duration. These findings contrast with those from a smaller study on children with asthma, in which improvements in FEV_1_ and FEV_1_/FVC were observed after SLIT.^36^ In another pediatric study, no improvement in lung function parameters (FEV_1_ and FEV_1_/FVC) were observed after SLIT for 6 months, although bronchial hyperreactivity was significantly reduced.^37^

We observed that Feno, a marker of airway inflammation, increased after treatment in children with SLIT duration of <4 months, but not in those with SLIT duration of >4 months. Similar results were reported by Ai et al, who found a significant reduction in Feno in 71 children with asthma treated with SLIT.^36^

The strengths of this study include the large dataset with detailed register-based clinical assessment of children with asthma receiving SLIT. We acknowledge that a control group of children with asthma not receiving SLIT therapy would have been useful. However, this was not possible in this dataset that is based on real-life data. Other limitations include lack of data on asthma medication, therapy adherence, disease severity, family history of atopy, disease exacerbation, other allergies, or environmental exposure. Furthermore, we have missing data from several clinical assessments, we lack posttreatment clinical assessment from 48% of the study population, and we do not know whether children who discontinued SLIT had begun subcutaneous immunotherapy. Another limitation is that SLIT duration was based on either the number of prescriptions (duration of >4 months) or the number of tablets (durations of >12 and >24 months), not on actual time intervals. We believe that these latter limitations will tend to yield an underestimation of the observed effects, and that our results are therefore nevertheless valid.

In summary, we have shown age and sex differences in children with asthma receiving SLIT and described the children’s clinical characteristics. We found that only a minority of children had SLIT duration of >2 years and that SLIT duration of >4 months was associated with having parents with higher education. Furthermore, SLIT duration of >12 months was inversely associated with uncontrolled asthma, but no associations between treatment duration and change in lung function were observed. Further studies are still needed to clarify the effect of SLIT on asthma in children, and actions are urgently needed to improve treatment persistence in children receiving SLIT.

## Disclosure statement

Financial support was provided by the 10.13039/501100004359Swedish Research Council (2018-02640 and 2023-02327), the Strategic Research Program in Epidemiology at 10.13039/501100004047Karolinska Institutet, the 10.13039/501100003793Swedish Heart-Lung Foundation (20210416, 20230629, and 20240974), the 10.13039/501100010234Swedish Asthma and Allergy Association Research Fund (2021-0035 and 2024-0010), Region Stockholm (ALF projects [RS2022-06]), the Foundation “Frimurare Barnhuset in Stockholm,” and the Konsul Th. C. Bergh’s Foundation (240018).

Disclosure of potential conflict of interest: J. R. Konradsen reports, outside the present report, advisory board fees from Novartis and ALK; and institutional fees from Regeneron Pharmaceuticals. C. Stridsman reports, outside the present report, personal fees from AstraZeneca and GSK; and institutional fees from Chiesi and TEVA. M. Ingemansson reports personal fees from ALK. The rest of the authors declare that they have no relevant conflicts of interest.
