# Supplementary material for: Sublingual immunotherapy in children with asthma: A population-based register study
Source: J Allergy Clin Immunol Glob. 2025 Sep 30;5(1):100574. doi: 10.1016/j.jacig.2025.100574 (PMC12581690; doi:10.1016/j.jacig.2025.100574)
Supplement: Supplementary Tables [file mmc1.docx]

**Online repository Table 1. Sociodemographic and clinical characteristics of children with post-treatment assessments in SNAR.**

|  | **N** | **Post-treatment asessment**  **n=782** |
| --- | --- | --- |
|  |  |  |
| Female, n (%) | 782 | 229 (29.3) |
| Male, n (%) | 782 | 553 (70.7) |
| Younger child, n (%) | 782 | 244 (31.1) |
| Adolescent, n (%) | 782 | 538 (68.8) |
| Parent with higher education, n (%) | 782 | 577 (73.8) |
| No parent with higher education, n (%) | 782 | 205 (26.2) |
| ACT score, mean (SD) | 542 | 21.6 (3.4) |
| ACT score ≤ 19, n (%) | 542 | 102 (18.8) |
| ACT score > 19, n (%) | 542 | 440 (81.2) |
| FEV_1_ (%), mean (SD) | 367 | 93.0 (15.3) |
| BDR FEV_1_ (%), mean (SD) | 258 | 4.7 (13.7) |
| FENO, ppb, mean (SD) | 245 | 21.1 (19.1) |

ACT: asthma control test; SD: standard deviation; FEV_1_, forced expiratory volume in 1 s; FVC, forced vital capacity; BDR, bronchodilator response; FENO: fraction of exhaled nitric oxide; ppb: parts per billion.

**Online repository Table 2. Associations between the exposures treatment duration and the outcome post-treatment clinical characteristics, stratified by type of treatment.**

| **Grazax** | **Treatment duration > 4 months vs. treatment duration < 4 months** | | | **Treatment duration > 12 months vs. treatment duration < 4 months** | | | **Treatment duration > 24 months vs. treatment duration < 4 months** | | |
| --- | --- | --- | --- | --- | --- | --- | --- | --- | --- |
|  | **n** | **OR_crude_** | **OR_adj_** | **n** | **OR_crude_** | **OR_adj_** | **n** | **OR_crude_** | **OR_adj_** |
| Uncontrolled asthma | 398 | 0.61 (0.32–1.16) | 0.60 (0.30–1.17) | 278 | **0.48 (0.24–0.97)** | **0.46 (0.22–0.96)** | 173 | 0.59 (0.27–1.26) | 0.59 (0.26–1.33) |
|  | **n** | **B_crude_** | **B_adj_** | **n** | **B_crude_** | **B_adj_** | **n** | **B_crude_** | **B_adj_** |
| ACT score, mean (SD) | 398 | -0.14 (-1.1–0.8) | -0.19 (-1.2–0.8) | 278 | 0.15 (-0.9–1.2) | 0.16 (-0.9–1.2) | 173 | 0.02 (-1.1–1.1) | -0.04 (-1.3–1.2) |
| FEV_1_ (%), mean (SD) | 300 | 2.45 (-1.9–6.8) | 2.87 (-2.1–7.8) | 220 | 2.03 (-2.7–6.8) | 2.67 (-3.7–9.1) | 137 | 1.04 (-3.4–5.5) | -0.02 (-4.4–4.4) |
| FEV_1_/FVC, mean (SD) | 280 | 0.03 (0.0–0.1) | 0.02 (0.0–0.1) | 200 | 0.03 (0.0–0.1) | 0.03 (0.0–0.1) | 120 | 0.02 (0.0–0.1) | 0.02 (0.0–0.1) |
| BDR FEV_1_ (%), mean (SD) | 215 | -6.35 (-17.2–4.5) | -5.61 (-15.2–3.8) | 155 | -6.44 (-17.4–4.5) | -5.04 (-13.2–3.1) | 97 | -6.48 (-17.5–4.6) | -3.93 (-11.0–3.1) |
| FENO, ppb, mean (SD) | 172 | -8.2 (-19.4–3.1) | -8.2 (-18.4–2.1) | 122 | -9.21 (-20.6–2.2) | -9.11 (-19.5–1.2) | 75 | -7.57 (-19.6–4.5) | -6.9 (-17.0–3.1) |

| **Itulazax** | **Treatment duration > 4 months vs. treatment duration < 4 months** | | | **Treatment duration > 12 months vs. treatment duration < 4 months** | | | **Treatment duration > 24 months vs. treatment duration < 4 months** | | |
| --- | --- | --- | --- | --- | --- | --- | --- | --- | --- |
|  | **n** | **OR_crude_** | **OR_adj_** | **n** | **OR_crude_** | **OR_adj_** | **n** | **OR_crude_** | **OR_adj_** |
| Uncontrolled asthma | 212 | 0.80 (0.40–1.62) | 0.79 (0.38–1.62) | 132 | 0.82 (0.37–1.84) | 0.81 (0.35–1.87) | 78 | 1.05 (0.32–3.40) | 0.90 (0.26–3.12) |
|  | **n** | **B_crude_** | **B_adj_** | **n** | **B_crude_** | **B_adj_** | **n** | **B_crude_** | **B_adj_** |
| ACT score, mean (SD) | 212 | -0.28. (-1.3–0.8) | -0.31 (-1.4–0.8) | 132 | -0.19 (-1.4–1.0) | -0.07 (-1.4–1.2) | 78 | -0.25 (-1.9–1.4) | -0.01 (-1.7–1.6) |
| FEV_1_ (%), mean (SD) | 68 | 10.1 (-9.7–30.0) | 12.7 (-11.1–36.6) | 61 | 2.4 (-6.2–11.0) | 1.8 (-7.5–11.1) | **54** | **12.1 (7.1–17.0)** | **10.6 (4.7–16.5)** |
| FEV_1_/FVC, mean (SD) | 50 | 0.00 (-0.1–0.1) | 0.00 (-0.1–0.1) | 47 | 0.02 (0.0–0.1) | 0.02 (0.0–0.1) | 47 | 0.03 (0.0–0.1) | 0.02 (0.0–0.1) |
| BDR FEV_1_ (%), mean (SD) | 38 | -5.53 (-17.6–6.5) | -5.40 (-28.1–17.3) | 36 | -3.22 (-14.6–8.2) | 7.83 (-2.5–18.1) | 36 | -3.2 (-14.6–8.2) | 7.8 (-2.5–13.8) |
| FENO, ppb, mean (SD) | 93 | -4.3 (-16.4–7.8) | -2.25 (-13.9–9.3) | 60 | -4.9 (-18.6–8.9) | -5.3 (-17.2–6.6) | 33 | -10.4 (-24.5–3.8) | -8.7 (-24.3–6.8) |

| **Acarizax** | **Treatment duration > 4 months vs. treatment duration < 4 months** | | | **Treatment duration > 12 months vs. treatment duration < 4 months** | | | **Treatment duration > 24 months vs. treatment duration < 4 months** | | |
| --- | --- | --- | --- | --- | --- | --- | --- | --- | --- |
|  | **n** | **OR_crude_** | **OR_adj_** | **n** | **OR_crude_** | **OR_adj_** | **n** | **OR_crude_** | **OR_adj_** |
| Uncontrolled asthma | 145 | **0.39 (0.16–0.93)** | **0.35 (0.14–0.87)** | 110 | **0.25 (0.08–0.81)** | **0.23 (0.08–0.80)** | 83 | 0.27 (0.06–1.27) | 0.25 (0.05–1.23) |
|  | **n** | **B_crude_** | **B_adj_** | **n** | **B_crude_** | **B_adj_** | **n** | **B_crude_** | **B_adj_** |
| ACT, mean (SD) | 145 | 0.23 (-0.9–1.4) | 0.37 (-0.8–1.5) | 110 | 0.41 (-0.8–1.6) | 0.50 (-0.8–1.8) | 83 | 0.56 (-0.7–1.8) | 0.60 (-0.8–2.0) |
| FEV_1_ (%), mean (SD) | 119 | -0.50 (-5.2–4.2) | -1.15 (-5.8–3.5) | 92 | -3.22 (-8.3–1.9) | -3.7 (-8.7–1.3) | 70 | -3.8 (-10.1–2.4) | -4.5 (-10.6–1.5) |
| FEV_1_/FVC, mean (SD) | 116 | 0.00 (0.0–0.0) | 0.00 (0.0–0.0) | 88 | 0.00 (0.0–0.0) | 0.00 (0.0–0.0) | 66 | 0.00 (-0.1–0.0) | 0.00 (-0.1–0.0) |
| BDR FEV_1_ (%), mean (SD) | 92 | -5.6 (-16.7–5.6) | -4.0 (-12.4–4.4) | 68 | -5.3 (-16.8–6.3) | -3.7 (-17.0–5.7) | 51 | -3.2 (-16.0–9.4) | -1.7 (-12.0–9.1) |
| FENO, ppb, mean (SD) | 61 | -6.7 (-19.5–6.0) | -6.0 (-17.9–6.0) | 53 | -8.4 (-21.2–4.3) | -6.4 (-18.2–5.3) | 36 | -11.8 (-25.7–2.1) | -10.1 (-22.1–1.9) |

OR: odds ratio; ACT: asthma control test; SD: standard deviation; FEV_1_, forced expiratory volume in 1 s; FVC, forced vital capacity; BDR, bronchodilator response; FENO: fraction of exhaled nitric oxide; ppb: parts per billion.

*Uncontrolled asthma and ACT were adjusted for sex and parental education. FEV1, FEV1/FVC and BDR were adjusted for parental education. FENO was adjusted for sex, age group, and parental education.
